# A phospho-harmonic orchestra plays the NLRP3 score

**DOI:** 10.3389/fimmu.2023.1281607

**Published:** 2023-11-03

**Authors:** Frédéric Bornancin, Carien Dekker

**Affiliations:** ^1^ Immunology Department, Novartis Biomedical Research, Basel, Switzerland; ^2^ Discovery Sciences Department, Novartis Biomedical Research, Basel, Switzerland

**Keywords:** NLRP3, inflammasome, phosphorylation, kinase, phosphatase

## Abstract

NLRP3 is a prototypical sensor protein connecting cellular stress to pro-inflammatory signaling. A complex array of regulatory steps is required to switch NLRP3 from an inactive state into a primed entity that is poised to assemble an inflammasome. Accumulating evidence suggests that post-translational mechanisms are critical. In particular, phosphorylation/dephosphorylation and ubiquitylation/deubiquitylation reactions have been reported to regulate NLRP3. Taken individually, several post-translational modifications appear to be essential. However, it remains difficult to understand how they may be coordinated, whether there is a unique sequence of regulatory steps accounting for the functional maturation of NLRP3, or whether the sequence is subject to variations depending on cell type, the stimulus, and other parameters such as the cellular context. This review will focus on the regulation of the NLRP3 inflammasome by phosphorylation and dephosphorylation, and on kinases and phosphatases that have been reported to modulate NLRP3 activity. The aim is to try to integrate the current understanding and highlight potential gaps for further studies.

## 1 Introduction

NLRP3 (also known as cryopyrin or NALP3) is an inflammasome sensor that can be activated in response to a wide variety of stimuli, including both pathogen-associated and damage associated molecules (aka PAMPs, DAMPs) such as ATP, the potassium ionophore nigericin and lysosome-disruptive agents like cholesterol, silica, or monosodium urate (MSU) crystals, and by glycolytic changes as well as changes in the mitochondrial electron-transport chain (1–8). Recognition of DAMPs and PAMPs leads to NLRP3 conformational change and oligomerization, and eventually to recruitment of ASC and pro-caspase 1 to form the inflammasome. This triggers the dimerization, auto-processing, and activation of caspase-1, which drives cleavage of pro-IL-1β and pro-IL-18, leading to IL-1β and IL-18 secretion, as well as cleavage of Gasdermin D, which can result in inflammatory form of cell death known as pyroptosis.

For a long time, activation of the NLRP3 inflammasome was described as a two-step process: A first step, called “priming”, leading to transcriptional up-regulation of inflammasome genes including those encoding pro-IL-1β and NLRP3 itself, and a second step called “activation”, featuring assembly of the inflammasome components. In 2012, two studies (9, 10) provided the first evidence that a faster regulation, independent from elevation of protein levels, is involved in the “priming” process. Since then, several studies have contributed a body of evidence showing that additional layers of regulation take place after translational “priming” for “licensing” the NLRP3 protein. In this regard, several post-translational modifications (PTMs), such as ubiquitylation/de-ubiquitylation (11–18), SUMOylation (19–21), ISGylation (22), acetylation (23), nitrosylation (24–26), and phosphorylation/de-phosphorylation reactions are now known to be required to license NLRP3 for activation. The later will be the focus of this review.

Many kinases are reported to regulate NLRP3. In several instances, acceptor phospho-sites in NLRP3 were identified, and phosphatases acting at some of the phosphorylated sites have been proposed. Different strategies were used for producing phospho-NLRP3, which may diversely and only partially reflect the actual physiological process. In fact, there is still very little understanding of the overall phospho-modulatory process and several questions have remained unanswered. Are all reported post-translational modifications genuine sites of regulation? Which are the critical ones? Are some sites redundant? What is the sequence of events? Could PTMs be subject to variations, according to, *e.g.*, stimulus, cell-type, cellular context, or genetic polymorphisms?

This review will summarize the phospho-sites identified to date as if they would be notes in the NLRP3 “score”; the kinases and phosphatases that are reported to regulate NLRP3 activation are represented as orchestra players that are able to read and play the score. A potential scenario of how the phospho-regulatory process of NLRP3 may be integrated, *i.e.*, how the score may be played, is presented, informed by recent structural insights.

## 2 Reading the NLRP3 score with the phospho-orchestra players

**Figure 1:**
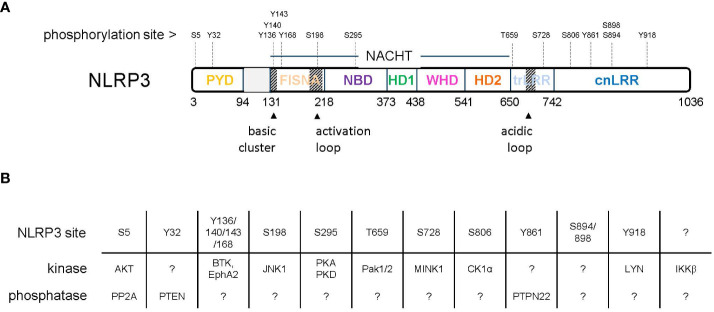
**(A)** Domain organization of NLRP3 with indicated phosphorylation sites. **(B)** Reported kinases and phosphatases acting on NLRP3.

**Figure 2:**
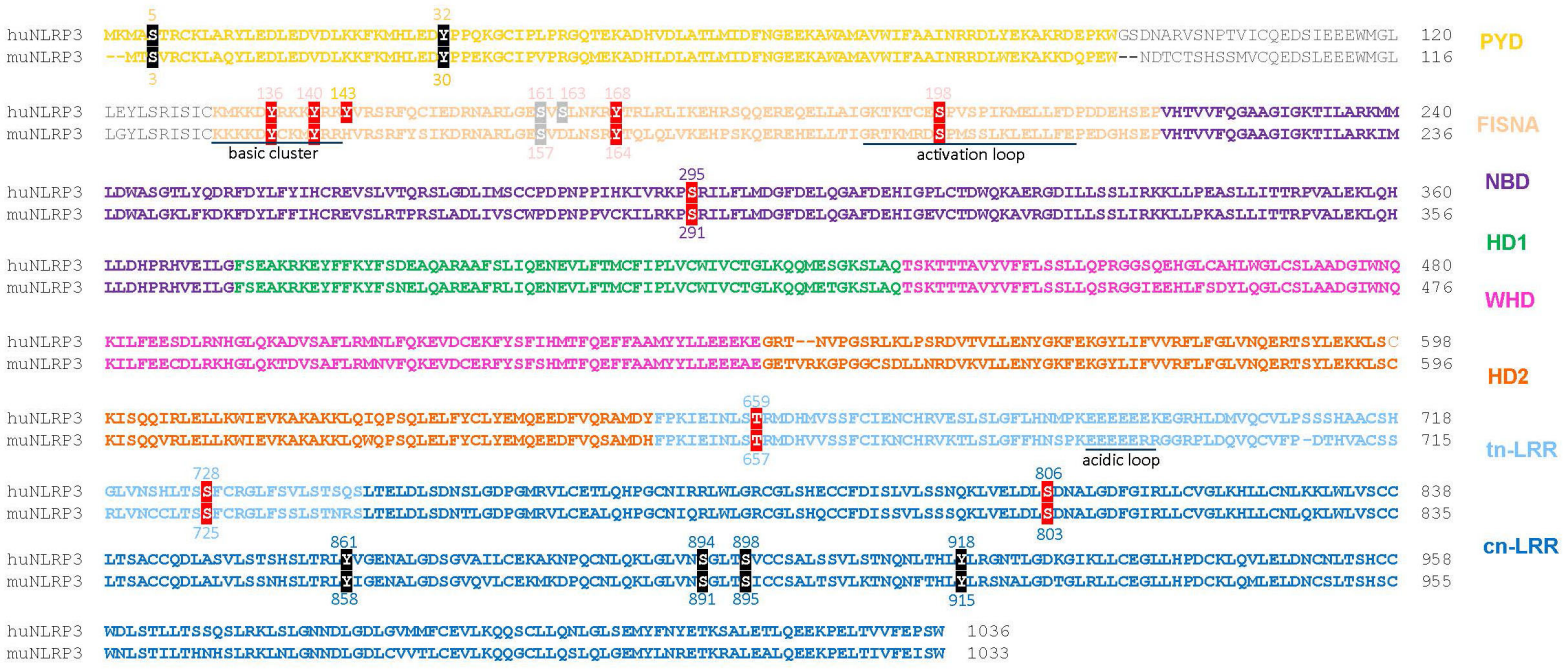
Sequence alignment of human NLRP3 (Q96P20 alias NP_004886) and mouse NLRP3 (Q8R4B8 alias NP_665826) with color-coded domains. Reported phosphorylation sites are boxed with colors according to the impact of phosphorylation: inhibitory (black), stimulatory (red), undefined (grey). PYD, pyrin domain; FISNA, fish-specific NACHT-associated; NBD, nucleotide binding domain; HD 1 and 2, histidine-aspartate domains 1 and 2, involved in phosphohydrolase activity; WHD, winged helix domain; LRR, leucine-rich repeats; trLRR, transitional LRR; cnLRR, canonical LRR.

A first read-through of the NLRP3 score will provide a two-dimensional sequence scan of the protein from N- to C-terminus, pointing out the part to play (NLRP3 subdomain) and the bars with an identified phospho-site. It will also state which leading instrument in the kinase and phosphatase sections may play the phospho-notes in the score (**Figures 1**, **2**).

**Part to play:** N-terminal part of PYD domain

**Bar:** Serine 5 *mouse: Serine 3*

**Kinase section lead:** Protein kinase B (AKT)

**Phosphatase section lead:** Protein Phosphatase 2A (PP2A)

Stutz et al. (27) used an LPS-treated murine line transduced with FLAG-NLRP3 to match endogenous NLRP3 levels. Immunoprecipitated NLRP3 was resolved, digested, and submitted to mass spectrometry analysis, leading to the identification of three conserved phosphorylated residues S5, S161, and S728. When mutagenized, only the S5 residue appeared critical. The phosphomimic S5D protein variant allowed neither for NLRP3-induced ASC speck formation nor for IL-1β secretion whereas there was limited impairment of these two readouts with the S5A NLRP3 variant. Furthermore, in transfected 293T cells, self-assembly of NLRP3 was abrogated by the S5D mutation. Thus, self-assembly as well as assembly with ASC is prevented by phosphorylation of S5. Then, using the protein phosphatase inhibitor okadaic acid together with knockout approaches in immortalized macrophages (iMOs), the protein phosphatase PP2A was shown to be involved in dephosphorylation of the S5 site, allowing for activation of NLRP3. The importance of PP2A in controlling NLRP3 activation has been further illustrated in recent work with human and mouse macrophages pointing to a role downstream of multiple TLRs (28). Another report suggested that PP2A activity may be downregulated by Bruton’s tyrosine kinase (BTK), which is recruited to NLRP3 following LPS stimulation and prevents dephosphorylation of S5. NLRP3 activation relieves inhibition of PP2A by triggering the dissociation of BTK from NLRP3 (29).

Recent progress based on a comprehensive set of experiments with the AKT inhibitor MK2206, the AKT activator SC-79 and genetic knock down has established that AKT is the kinase involved in phosphorylation of S5 (30). Immunoprecipitation and mutagenesis studies showed direct and constitutive interaction of NLRP3 with AKT and suggested that the LRR domain of NLRP3 and the kinase domain of AKT are involved. Direct phosphorylation of S5 by AKT was evidenced using an *in vitro* phosphorylation assay using immuno-precipitated AKT and HEK293T cell lysates expressing NLRP3. Next, cellular extracts and confocal imaging showed that AKT kinase inhibition leads to enhanced ASC oligomerization after LPS ± nigericin stimulation of BMDMs. The levels of NLRP3 were reduced after stimulation in THP1 cells when AKT was knocked down. Thus, AKT could have a dual and complementary role during NLRP3 activation by preventing NLRP3 degradation while inhibiting its oligomerization/inflammasome assembly process. Degradation of NLRP3 requires ubiquitin-dependent mechanisms involving E3 ligases such as TRIM31 (18). By using a set of experiments in THP-1 cells, modulating AKT and TRIM31 levels, and HEK293T cells transfected with NLRP3 and TRIM31 WT and variants, the authors showed that AKT stabilizes NLRP3 by phosphorylating S5, which blocks ubiquitylation of K496, thus preventing ubiquitin-proteasome degradation of NLRP3.

In conclusion, this study suggests a dual role of AKT in the regulation of NLRP3. Upon priming with LPS, AKT is activated and phosphorylates S5 in NLRP3 to prevent TRIM31-mediated NLRP3 ubiquitylation at K496 and subsequent degradation while prohibiting NLRP3 oligomerization in the absence of an activation signal. When activation is triggered, it leads to PP2A-mediated dephosphorylation of NLRP3-S5, which allows for NLRP3 inflammasome assembly. This dephosphorylation event also likely leads to TRIM31-mediated ubiquitylation and subsequent degradation of NLRP3, thereby controlling excessive pro-inflammatory signaling.

Interestingly, available data on the regulation of NLRP3 by S5 phosphorylation are compatible with early work showing the importance of a short stretch of amino-acids at the N-terminus of NLRP3 including S5 (2-KMASTR-7) both for recruitment to mitochondria via the antiviral signaling protein MAVS, and for activation (31). The sub-cellular aspect of NLRP3 regulation will be addressed later in this review.

**Part to play:** PYD domain

**Bar:** Tyrosine 32 *mouse: Tyrosine 30*

**Kinase section lead:** unknown

**Phosphatase section lead:** Phosphatase and Tensin homolog (PTEN)

By using recombinant FLAG-tagged NLRP3 immunoprecipitated after expression in HEK 293T cells, Huang et al. (32) identified five phosphorylation sites: Y32, S163, T193, T195, S216. Co-expression of PTEN completely abrogated the phosphorylation of Y32 but had only partial or no impact on the phosphorylation of the other sites. Knocking down PTEN expression in THP-1 cells inhibited NLRP3 activation induced by nigericin, MSU, or ATP. These effects were dependent on the protein-phosphatase activity of PTEN. The critical role of PTEN was then illustrated by reconstitution experiments in *Nlrp3*-knockout BMDMs showing that the presence of *Pten* is indispensable for WT NLRP3 to restore inflammasome activation. The PTEN-dependent dephosphorylation of Y32 was demonstrated using an anti-phosphospecific antibody. Priming BMDMs with LPS induced phosphorylation at Y32, which was completely dephosphorylated upon subsequent activation with nigericin. Dephosphorylation did not occur in *Pten*-knockout BMDMs. The critical role of dephosphorylation at Y32 during inflammasome activation was further evidenced by reconstitution of *Nlrp3*-knockout BMDMs with NLRP3^Y32E^, and by the isolation of BMDMs from knock-in mice harboring the *Nlrp3* ^Y30E^ allele, which both showed deficient inflammasome activation upon activation with nigericin, MSU, or ATP.

The kinase responsible for the PTEN-sensitive phosphorylation of Y32 in NLRP3 has yet to be reported.

**Part to play:** PYD/NACHT interdomain

**Bar:** Tyrosine 136 *mouse: Tyrosine 132*

**Kinase section lead:** Ephrin type-A receptor 2 (EphA2)

**Phosphatase section lead:** unknown

The Ephrin type-A receptor 2 (EphA2) transmembrane tyrosine kinase is selectively expressed in airway epithelial cells in human and mouse. Knock-down of EphA2 in epithelial cells was shown to up-regulate IL-1β and IL-18 production after stimulation with the RNA virus reovirus (33). In keeping with this, Ephrin A1-mediated down-regulation of EphA2 led to enhanced production of IL-1β following stimulation of epithelial cells with reovirus. Furthermore, a murine model of reovirus infection conducted with wild-type and *EphA2*-knockout mice confirmed the role of EphA2 in the regulation of inflammasome activation and the resulting lung inflammation.

Pull-down experiments in airway epithelial cells indicated that EphA2 interacts with the LRR domain of NLRP3, but only after the cells have been infected with reovirus. Immunofluorescence imaging revealed endogenous NLRP3 colocalized with EphA2 in the cytosol, and this colocalization was enhanced with reovirus infection. Direct phosphorylation of NLRP3 by EphA2 was demonstrated by cellular co-expression studies, *in vitro* kinase assays with pulled-down proteins, and endogenously using airway epithelial cells from control or *EphA2*-knockout mice primed with LPS or infected with reovirus. Y132 was identified as the most critical phosphorylation site among five Tyrosine residues predicted as phospho-sites with high confidence scores by the NetPhos prediction server (Y132, Y164, Y251, Y570, and Y589). Deficiency in EphA2, or Y132F mutation of NLRP3, resulted in increased inflammasome activation (number of cells with an ASC speck, IL-1β cleavage), which led the authors propose Y132 (Y136 in human) as the EphA2 phosphorylation site in NLRP3. This, however, remains to be more firmly evidenced.

**Part to play:** PYD/NACHT interdomain

**Bar:** Tyrosines 136, 140, 143, 168 *mouse: Tyrosines 132, 136, 164*

**Kinase section lead:** Bruton tyrosine kinase (BTK)

**Phosphatase section lead:** unknown

Several reports suggest a role for BTK in the regulation of the NLRP3 inflammasome (29, 34–36). Peripheral blood mononuclear cells (PBMCs) deficient in BTK displayed impaired production of IL-1β following induction by ATP or nigericin (36). Nigericin induced BTK phosphorylation within minutes in PMA-primed THP-1 or LPS-primed human monocyte-derived macrophages (36). In both human and murine primary immune cells, BTK could interact with NLRP3 independently of stimulation (35). Stimulation with nigericin or MSU crystals induced tyrosine phosphorylation of NLRP3, which did not occur in the absence of BTK. The co-expression of a kinase-dead variant of BTK together with NLRP3 in a reconstituted system in HEK293 cells allowed NLRP3-BTK interactions but failed to induce NLRP3 tyrosine phosphorylation. However, direct phosphorylation of NLRP3 by BTK is still lacking robust evidence as the work was conducted *in vitro* or in reconstituted HEK293 cellular system, which may not recapitulate inflammasome biology in physiological cellular systems. In addition, the strict requirement for BTK kinase activity remains questionable as experiments made use of 60 μM of ibrutinib (35, 36), a compound known to lack selectivity for BTK at this concentration (37).

By co-expressing various subdomains of NLRP3 together with BTK in HEK293 cells, Bittner et al. (35) identified four BTK-dependent phosphorylation sites (Y136, Y140, Y143, Y168) all located in the linker region (aa 94-219) between the PYD (pyrin domain) and the NACHT (NAIP, CIITA, HET-E and TEP) domains, and highly conserved throughout evolution. They showed that NLRP3 variants with Y>E mutations in this region display reduced binding to phosphatidyl inositol 4-phosphate (PI4P), a phospholipid enriched at the trans-Golgi network (TGN). In BMDMs, absence of BTK suppressed NLRP3 oligomerization and reduced the abundance of high-order oligomers of ASC (of note, the effect of high ibrutinib concentrations was less clear on the latter). Size exclusion chromatography of cell lysates further showed a slight shift of NLRP3 to lower MW complexes. Nigericin-induced ASC speck formation was diminished in the absence of BTK. BTK knockout also modestly decreased ASC specks in ASC-mCerulean iMacs after nigericin treatment, but a strong reduction was observed when the lysosomal damaging agent LeuLeu-O-Me was used instead of nigericin. NLRP3-deficient iMacs reconstituted with either WT NLRP3 or its Y(136,140,143,168)F variant were used to probe IL-1β production in response to inflammasome activation. Consistent with the oligomerization data, Y(136,140,143,168)F mutation modestly decreased nigericin-induced IL-1β and abrogated imiquimod-induced IL-1β.

Collectively, BTK is emerging as a potential kinase for regulating the NLRP3 inflammasome during activation, by modification of a protein motif involved in subcellular localization and inflammasome assembly. The partial effects observed upon nigericin treatment suggest that this pathway of activation operates independently of BTK. Why stimulation by other means, *e.g.*, LeuLeu-O-Me, or imiquimod appears more dependent on BTK suggests the existence of an intriguing second pathway for NLRP3 activation that deserves further elucidation (see also section 5 below). In addition, whether the four tyrosine residues identified in this work are direct substrates of BTK under physiological conditions awaits more definitive evidence.

**Part to play:** PYD/NACHT interdomain

**Bar:** Serine 198 *mouse: Serine 194*

**Kinase section lead:** cJun N-terminal kinase 1 (JNK1)

**Phosphatase section lead:** unknown

S198 lies in a loop at the interface between the NACHT and LRR domains, recently coined the “activation loop” (38). In their pioneering work, Song et al. (39) used a reconstitution assay based on co-transfection of NLRP3, ASC, pro-caspase-1, and pro-IL-1β in HEK293T cells, which enabled inflammasome activation. NLRP3 was immunoprecipitated and analyzed by LC/MS, leading to identification of phosphorylated S198. Mutagenesis studies then showed no activity for the S198A NLRP3 variant, whereas the phospho-mimetic S198D or -E variants showed increased activity *vs*. WT in their reconstitution assay. In immortalized mouse bone marrow-derived macrophages (iBMDMs) with endogenous *Nlrp3* knock-down, stable transfection of the S194A variant showed abrogated NLRP3 activation following stimulation with nigericin, ATP or MSU, by comparison to WT NLRP3 controls. Similar findings were obtained with *ex vivo* BMDMs from S194A NLRP3 knock-in mice. Subjecting these mice to an MSU-induced peritonitis model and to an LPS-induced sepsis model showed that S194 phospho-mutation reduced IL-1β levels, reduced peritoneal extrudate cell numbers, and conferred higher resistance to sepsis, thus providing compelling evidence for the importance of this serine residue for mouse NLRP3 inflammasome function. Phosphorylation at S198 occurred early during the priming step (*e.g.*, 10 min after LPS stimulation). Combining the CAPS (Cryopyrin-associated periodic syndromes) variant T346M, which needs only priming for activation, together with a S198A mutation abolished LPS-induced inflammasome activation. However, the S198D NLRP3 mutant protein still required priming for activation, suggesting this key phosphorylation does not recapitulate the whole priming process.

The authors then used a combination of prediction tool analyses, inhibitory approaches (the JNK1 inhibitor SP600125, the unspecific JNK1 activator anisomycin, and genetics) as well as *in vitro* kinase assays to identify JNK1 as the kinase responsible for NLRP3 phosphorylation at S198. Finally, they showed that phosphorylation of S198 by JNK1 allows for de-ubiquitylation of NLRP3 by BRCC3, an essential step for NLRP3 inflammasome activation.

Unpublished data from our laboratory have confirmed that the S198D mutant protein drives stronger inflammasome activation compared to WT NLRP3 (ASC speck formation, caspase-1 cleavage) based on a reconstitution assay related to that published by Song et al. (39). In our hands, the S198A mutant protein led to similar activation compared to WT NLRP3, in line with observations by Dufies et al. (40). In addition, recent work by Wu et al. (41) showed that crystalline silica particles can elicit S198 phosphorylation of NLRP3 in airway epithelial cells, confirming the relevance of this phosphorylation site. The importance of JNK1 in licensing NLRP3 activation has received further support with the evidence that IRAK1-dependent supramolecular organizing centers (SMOCs) can form upon multi-TLR stimulation and rely on JNK kinase function to facilitate licensing of the inflammasome (42).

**Part to play:** NACHT domain

**Bar:** Serine 295 *mouse: Serine 291*

**Kinase section lead #1:** Protein kinase A (PKA)

**Kinase section lead #2:** Protein kinase D (PKD)

**Phosphatase section lead:** unknown

In 2016, a study by Mortimer et al. addressed the long reported inhibitory effect of prostaglandins such as PGE2 on inflammasome activation (43), showing that PGE2 does not block transcriptional upregulation of NLRP3 and IL-1β but can prevent and block inflammasome activation induced by several agents (nigericin, ATP, silica, MSU) in BMDMs and THP-1 cells. The effect was dependent on the EP4 receptor and could be phenocopied by using forskolin and dibutyryl cAMP. Furthermore, forskolin could disassemble NLRP3/ASC complexes in reconstituted HEK293 cells. They showed that PKA mediates the cAMP effects by blocking the ATPase activity of NLRP3.

Using Prosite, they identified two potential PKA phosphorylation sites in NLRP3: S295 and S597 (also conserved in mouse). The S295A variant was the only one blocking forskolin-mediated inflammasome inhibition, as measured by ASC oligomerization, IL-1β release, and ATP hydrolysis. In fact, this mutation enhanced inflammasome activity triggered by cAMP. Of note, the D305G mutation, proximal to S295 and known to cause NOMID, the most severe manifestation of CAPS, had a similar phenotype. It is hypothesized that these CAPS-susceptible NLRP3 mutations might potentially resist control by PKA-induced phosphorylation (43, 44).

Further studies have confirmed a PKA-dependent inhibitory phosphorylation of NLRP3. Guo et al. (44) suggested that bile acids bind to receptors such as TGR5, leading to PKA-mediated phosphorylation of NLRP3 at S291 (mouse). The role of TGR5 in mediating the inhibitory effects of bile acids on the NLRP3 inflammasome has received additional support in a recent study by Liao et al. (45). In another study evaluating phosphatidyl-inositol dependent modulation of the NLRP3 inflammasome, the authors observed that increased incorporation of long acyl group chains in phosphatidyl-inositol results in S291 phosphorylation, likely dependent on PGE2-induced PKA signaling (46).

In addition to the PKA axis, a study by Zhang et al. provided novel insights into the key regulatory impact of phosphorylation at S295, this time by another kinase, protein kinase D (47). NLRP3 can associate with mitochondrial-associated ER membranes (MAMs) while PKD is actively recruited and activated at Golgi membranes upon sensing an increase in diacylglycerol (DAG). In BMDMs, in response to NLRP3 inflammasome activators, *e.g.*, nigericin, DAG levels increase at the Golgi and MAMs become adjacent to Golgi membranes, thus allowing for phosphorylation of NLRP3 by PKD. By using PKD inhibitors as well as mouse models of PKD deficiency, Zhang et al. uncovered the contribution of PKD enzyme activity for the release of active NLRP3 into the cytosol, capable of assembling with ASC. By ectopic expression of NLRP3 and PKD proteins (both NLRP3 WT and variant forms including point mutations at anticipated putative phosphorylation sites) they identified S293 within the nucleotide binding domain of NLRP3 (S295 in human) as the site sensitive to regulation by PKD. Confirmation of direct phosphorylation of endogenous NLRP3 by PKD has remained challenging because sensitive antibody tools are not available, and likely because the S291 (S293 in their report) can also be phosphorylated by PKA (see above). However, the importance of PKD in the regulation of NLRP3 was further highlighted in a recent study by Heiser et al. (48).

**Part to play:** transition-LRR domain

**Bar:** Threonine 659 *mouse: Threonine 657*

**Kinase section lead:** p21-activated kinase 1 and 2 (Pak1/2)

**Phosphatase section lead:** unknown

A recent study by Dufies et al. showed that the NLRP3 inflammasome can be triggered upon activation of Rho GTPases (*e.g.*, Rac2) by bacterial toxins and virulence factors in human and mouse macrophages (40). This is dependent on the activity of p21-activated kinases (Pak), particularly Pak1 but does not require LPS pre-treatment. In fact, NLRP3 can interact with Rac in the presence of active Pak1. Nigericin-induced NLRP3 activation in BMDMs was also dependent on Pak1 activity. An *in vitro* kinase assay using N-terminally GST-tagged full length recombinant wheat germ expressed human NLRP3 showed that Pak1 can directly phosphorylate NLRP3 and led to identification of three phosphorylated residues, S163, T659, and S198. Only T659 appeared critical in subsequent mutagenesis and reconstitution experiments.

**Part to play:** transition-LRR domain

**Bar: Serine 728** *mouse: Serine 725*

**Kinase section lead:** MSN kinase 1 (MINK1)

**Phosphatase section lead:** unknown

The PhosphoSitePlus database rated S728 as the most likely phosphorylated site in NLRP3 and there is evidence this site or possibly the nearby S735 can be phosphorylated (27, 39, 49). Zhu et al. (50) embarked on the search for the kinase responsible for phosphorylation at S728. They started with BMDMs from *Mink1* knockout mice and showed that the lack of Mink1 reduces the level of NLRP3 activation following various stimuli. Next, they used LPS-induced sepsis and alum-induced peritonitis models with these mice, adding evidence for reduced NLRP3-dependent inflammatory responses *in vivo*. MINK1 deficiency did not influence the transcript levels of the NLRP3 inflammasome components, including that of pro-IL1β, suggesting that MINK1 may regulate the NLRP3 activation step. Reconstitution experiments in BMDMs from *Mink1* knockout mice showed that the kinase domain of MINK1, not the regulatory domain is required to support inflammasome activation. Kinase activity of MINK1, however, only had a limited impact based on the study of the K54R MINK1 mutant. Pull-down experiments showed that the LRR domain of NLRP3 binds to MINK1, which is where S725 is located. *Mink1*-deficient BMDMs primed with LPS and stimulated with nigericin showed reduced phosphorylation of NLRP3. However, clear cut evidence for direct phosphorylation of S725 by MINK1 was not provided in this study. Phosphomimic variants of the S725 site (S725D, S725E) in NLRP3, expressed in *Nlrp3* deficient BMDMs could reconstitute inflammasome activation whereas the constitutive unphosphorylated S725A variant failed to signal in this assay, thereby showing the importance of phosphorylation of the S725 site. Reconstitution experiments in HEK293 cells suggested that phosphorylation at S725 is important for NLRP3 oligomerization. ROS are known to activate MINK1 and accordingly, scavenging them reduces MINK1 activity. This coincided with the inhibitory effect of ROS scavengers on IL-1β and caspase-1 activity in BMDMs, which was not observed in the absence of MINK1. Similar data were obtained in THP1 cells supporting the hypothesis that MINK1 may connect ROS production to the modulation of NLRP3 activation.

**Part to play:** canonical-LRR domain

**Bar:** Serine 806 *mouse: Serine 803*

**Kinase section lead:** Casein kinase 1-α (CSNK1A1)

**Phosphatase section lead:** unknown

Niu et al. (49) used mass spectrometry after ectopic expression of the LRR domain of NLRP3 in HEK293T cells together with optimized conditions to minimize deubiquitylation and proteolysis. This allowed for identification of the S806 phosphorylation site in huNLRP3, conserved across species. Expressing the S806A/S806D variants in NLRP3-deficient U937 cells led to partially/fully defective inflammasome activity, respectively, following priming with LPS and activation with nigericin (the effect also occurred with other priming agents such as Pam3CSK4 and stimuli like MSU or silica). The corresponding mutations in the mouse NLRP3 proteins (S803A/D) fully blocked NLRP3 function upon reconstitution of NLRP3-deficient mouse BMDMs and stimulation with nigericin or extracellular ATP. Further experiments in this mouse model demonstrated that S803D-NLRP3 cannot recruit ASC and fails to form the characteristic speck after LPS priming and activation by nigericin. In fact, the S806D (S803D) variants were also unable to recruit NEK7. Remarkably, this resulted in failure of the NLRP3 variants to recruit BRCC3. The S806D NLRP3 variant underwent K48-hyperubiquitylation, triggering its degradation. In keeping with functional deficiency of S803D NLRP3 at a cellular level, homozygous mice expressing this variant form were less sensitive to endotoxin shock, as seen in NLRP3-deficient animals.

Niu et al. then used a comprehensive panel of experiments leading to identification of Casein Kinase 1 alpha1 (CSNK1A1, CK1α) as the responsible kinase for phosphorylation of S806/S803. Phosphorylation of S806/S803 occurs during priming. Consistently, it was not phosphorylated in NLRP3 samples obtained from unchallenged expressing cells (51). Subsequent dephosphorylation of S806 during the activation step is required for NLRP3 function. Because this site appears critical in human and mouse under various stimulatory conditions it has emerged as a possible “universal checkpoint” for inflammasome assembly. Moreover, phosphorylation of this site appears to control subsequent phosphorylation at alternative serine residues because the S803A NLRP3 mutant did not react with an anti-phospho-serine antibody, in contrast to WT NLRP3. However, this remains to be further elucidated because experiments aiming at analyzing phosphorylation of S198, — another reported key Serine of NLRP3 that is phosphorylated during priming (see above), were not clearly conclusive.

This work provided information about the contribution of NEK7 to the process of NLRP3 activation. The fact that the NLRP3 S803A mutant can recruit NEK7 (and BRCC3) upon stimulation while displaying only low activity suggests NEK7 recruitment is not sufficient for NLRP3 activation. Contrary to S806A, the S806D mutant was unable to recruit NEK7 and was completely inactive. Thus, dephosphorylation of S806 is an obligatory step. Identification of the relevant phosphatase is a subject of continuing studies by this group.

**Part to play:** canonical-LRR domain

**Bar:** Tyrosine 861 *mouse: Tyrosine 858*

**Kinase section lead:** unknown

**Phosphatase section lead:** PTPN22

In THP-1 cells, PTPN22 was shown to be involved in NLRP3 activation (52, 53). Interaction between PTPN22 and NLRP3 was induced by NLRP3 stimuli in THP-1 cells, human PBMCs as well as primary mouse BMDCs, and NLRP3 was concomitantly dephosphorylated on tyrosine residues. Loss of PTPN22 did not alter basal NLRP3 phosphorylation and even enhanced the levels of phospho-tyrosine content upon activation. Conversely, upon expression of a GoF-autoimmunity-associated PTPN22 variant, basal and induced levels of phospho-tyrosine in NLRP3 were abolished. Mapping of the phosphorylated tyrosine residue targeted by PTPN22 took advantage of the comparison between NLRP3 splice variants as tyrosine phosphorylation of NLRP3 could be detected in full length NLRP3. This led the authors to hypothesize that Y861 (the only tyrosine residue specific to the long NLRP3 form) might be the candidate site, which was confirmed by using recombinant expression in HEK293T cells combined with site-directed mutagenesis, showing a complete loss of tyrosine phosphorylation when the Y861F mutant form was expressed by comparison to WT. Then, the authors used *Nlrp3*knockout BMDMs reconstituted with WT or Y861 mutant forms of NLRP3. Increased inflammasome activation was observed with a non-phosphorylated mutant form of NLRP3 compared with abrogated activity when a phospho-mimetic mutant was employed.

NLRP3 stimuli increased the phospho-tyrosine content of immunoprecipitated NLRP3 from autophagosomes whereas this signal decreased in whole cell lysates (54). In fact, only phosphorylated NLRP3 associated with phagophores, which suggests a mechanism for phosphorylation-induced degradation via autophagy.

**Part to play:** canonical-LRR domain

**Bar:** Serines 894, 898 *mouse: Serines 891, 895*

**Kinase section lead:** unknown

**Phosphatase section lead: **unknown

A recent study by Wang et al. (55) provided indirect evidence for phosphorylation of NLRP3 in a potential phosphodegron motif (NSGLTS) located in the LRR subdomain 8. Phosphorylation of the two serine residues S894 and S898 within this motif may be required for the binding of β-transducin repeat containing E3 ubiquitin protein ligase 1 (β-TrCP1), which they showed can bind NLRP3 and mediates its K27-ubiquitnation at Lys380, resulting in proteasomal degradation of NLRP3. In addition, the protein YAP, a key component of the Hippo pathway, which is involved in several cellular stress mechanisms to keep tissue homeostasis and organ size under control, competed with β-TrCP1 for binding to NLRP3, thereby alleviating proteasomal degradation to promote NLRP3 activation.

**Part to play:** canonical-LRR domain

**Bar: **Tyrosine 918 *mouse: Tyrosine 915*

**Kinase section lead: **Lyn

**Phosphatase section lead: **unknown

Tang et al. (56) noticed that NLRP3 in LPS-primed mouse BMDMs become tyrosine phosphorylated and ubiquitinated within minutes of stimulation with ATP or nigericin, a process that is fully blocked by the pan-Src PTK inhibitor Src-I1. Remarkably, no tyrosine phosphorylation was observed upon activation with MSU or silica, suggesting that this mechanism of NLRP3 regulation may pertain only to soluble stimuli. Pull-down experiments identified Lyn as the only protein tyrosine kinase associated with NLRP3. Tyrosine phosphorylation of NLRP3 was abrogated in *Lyn* ^-/-^ BMDMs and recovered upon expression of Lyn^WT^ but not upon expression of its inactive Lyn^Y397F^ variant. Furthermore, *Lyn^−/−^* BMDMs treated with LPS and then stimulated with ATP displayed reduced NLRP3 ubiquitylation and degradation and produced significantly higher IL-1β than WT BMDMs. Site directed mutagenesis of NLRP3 potential Lyn-targeted phosphorylated residues (predicted with NetPhos 3.1) in NLRP3 constructs used to reconstitute *Nlrp3^-/-^* BMDMs led to identification of Y918 as the phosphorylation site. *Lyn^-/-^* mice were highly susceptible to LPS-induced septic shock and died within the first 24h after LPS whereas blocking NLRP3 activity with MCC950 rescued the mice from lethality.

The process of NLRP3 regulation by Lyn remains to be clarified. The study of Tang et al. indicates that Lyn may constitutively interact with NLRP3, and that increased association may occur during activation of NLRP3. In their experiments, stimulation of the inflammasome with, *e.g.*, ATP, led to enhanced tyrosine phosphorylation whereas a previous report by Spalinger et al. (52, 53) suggested tyrosine phosphorylation during priming and subsequent de-phosphorylation during activation. Different lengths of priming with LPS may explain the discrepancies between the two studies.

**Part to play: **unresolved

**Bar: **unknown

**Kinase section lead: **Inhibitor of Kappa B kinase beta (IKKβ)

**Phosphatase section lead: **unknown

Transcriptional regulation of NLRP3 inflammasome components relies on canonical NF-κB. Consequently, inhibitors of IκKβ, the key kinase in this pathway, are effective blockers of inflammasome function. Besides, compelling evidence suggests IKKβ also plays a role during the activation phase of the NLRP3 inflammasome. Furthermore, the ubiquitin-ligase activity of TRAF6, a well-known regulator of IKKβ, contributes to non-transcriptional priming of NLRP3, by impacting on the inflammasome assembly process (57).

Nanda et al. (58) used BMDMs stimulated by short incubations with LPS to investigate non-transcriptional mechanisms and found that blocking IKKβ with pharmacological inhibitors or reducing its level by RNA interference could prevent LPS/ATP (or LPS/nigericin) induced NLRP3 activation. IKKβ is known to activate several kinases, such as the IKK-related kinases TBK1 and IKKε (59), COT kinase (aka Tpl2) (60, 61) and LRRK2. However, pharmacological or genetic inhibition of these kinases did not impact NLRP3 activation, implying a direct role of IKKβ in this process. The work by Nanda et al. (58), together with an independent study by Unterreiner et al. (62) has shown that blocking IKKβ reduces ASC speck formation in BMDM and THP-1 cells following stimulation with nigericin. Nanda et al. (58) used a proximity ligation assay in stimulated BMDMs and showed that IKKβ activity is required for approximation of NLRP3 to TGN38^+^ vesicles (58, 63, 64). Unterreiner et al. (62) focused on caspase-1 activation and showed that blocking IKKβ enzyme activity leads to stabilization of procaspase-1 by limiting its auto-proteolysis. A recent study has further elucidated the role of IKKβ in the NLRP3 activation process, namely for recruitment of NLRP3 to PI4P-containing vesicles (65). Importantly, this study revealed that priming of NLRP3 by IKKβ suppresses the dependency on NEK7 for activating NLRP3. Still, NEK7 was found to accelerate NLRP3 priming at early time points. Mechanistically, IKKβ primes NLRP3 by increasing the recruitment of NLRP3 to PI4P.

Recent immunoprecipitation studies suggested interaction between IKKβ and NLRP3 (66) but the identity of the IKKβ phosphorylation substrate remains to be elucidated. Some proteins, such as the inhibitor of Kappa B kinase (IκBα), the IKK-related kinases (TBK1 and IKKε), or Tpl2 and LRRK2 can be ruled out (58, 62); possible candidates might be proteins that facilitate interaction between NLRP3 and TGN38^+^ vesicles or, alternatively, proteins playing a role during oligomerization and assembly of the NLRP3 inflammasome components.

**Part to play:** unresolved

**Bar:** unknown

**Kinase section lead:** TANK-binding kinase 1 (TBK1), Inhibitor of Kappa B kinase epsilon (IKKε)

**Phosphatase section lead:** Protein phosphatase 2A (PP2A)

The work by Fischer et al. (28) described a “parking brake” mechanism at priming stage controlled by the catalytic activity of two related kinases, TANK-binding kinase 1 (TBK1) and I-kappa-B kinase epsilon (IKKϵ), which are activated by TLR ligation. Inhibitors of these kinases increased ASC speck formation in iBMDMs. TBK1/IKKe inhibitors could also block, at least partially, the suppressive effect of okadaic acid, used to inhibit PP2A, thereby suggesting TBK1/IKKe might counter the activating function of PP2A to keep NLRP3 from becoming inadvertently activated.

AKT is a well described substrate of TBK1. However, neither inhibition of AKT activity nor the mutagenesis of S5 in NLRP3 into Alanine (S5 is phosphorylated by AKT, see above) could prevent the binding of TBK1 to NLRP3. The binding of endogenous active-, phospho-TBK1 and endogenous NLRP3 could be measured by proximity ligation assay in iBMDMs, peaking at 30-60 min following LPS stimulation. TBK1/IKKe inhibition did not impact on the interaction between NLRP3 and NEK7, but it strongly increased ASC speck formation. While an S5A NLRP3 variant expressed in BMDMs was insensitive to AKT inhibition, it remained sensitive to TBK1/IKKe inhibition and to PP2A inhibition, suggesting that the regulation afforded by TBK1/IKKe and by PP2A goes beyond modulation of the S5 site.

## 3 Analyzing the NLRP3 score: structural implication of phospho-sites

Several recent cryo-electro-microscopy studies revealed that full length NLRP3 (mouse and human) can present as double-ring barrels or cages representing multimeric forms of NLRP3 (38, 51, 67). This occurs mainly via LRR-LRR interactions. The PYD is also believed to contribute to the barrel because its absence resulted in limited oligomerization. The top and bottom surfaces of the NLRP3 barrel display the poly basic region of the NACHT domains that provides a scaffold for NLRP3 membrane attachment. Adjacent NACHT domains barely interact with each other in the NLRP3 cage. However, they contribute by locking the structure, preventing self-oligomerization of the PYD and subsequent activation. These caged-type NLRP3 oligomers contain inactive NLRP3 protomers, hence are currently seen as a cellular form of storing NLRP3 prior to priming. Very recently, the structure of the fully assembled active NLRP3 inflammasome was reported (67) for the first time. This major achievement adds the final snapshot of NLRP3 structural states along the activation timeline.

**Figure 3:**
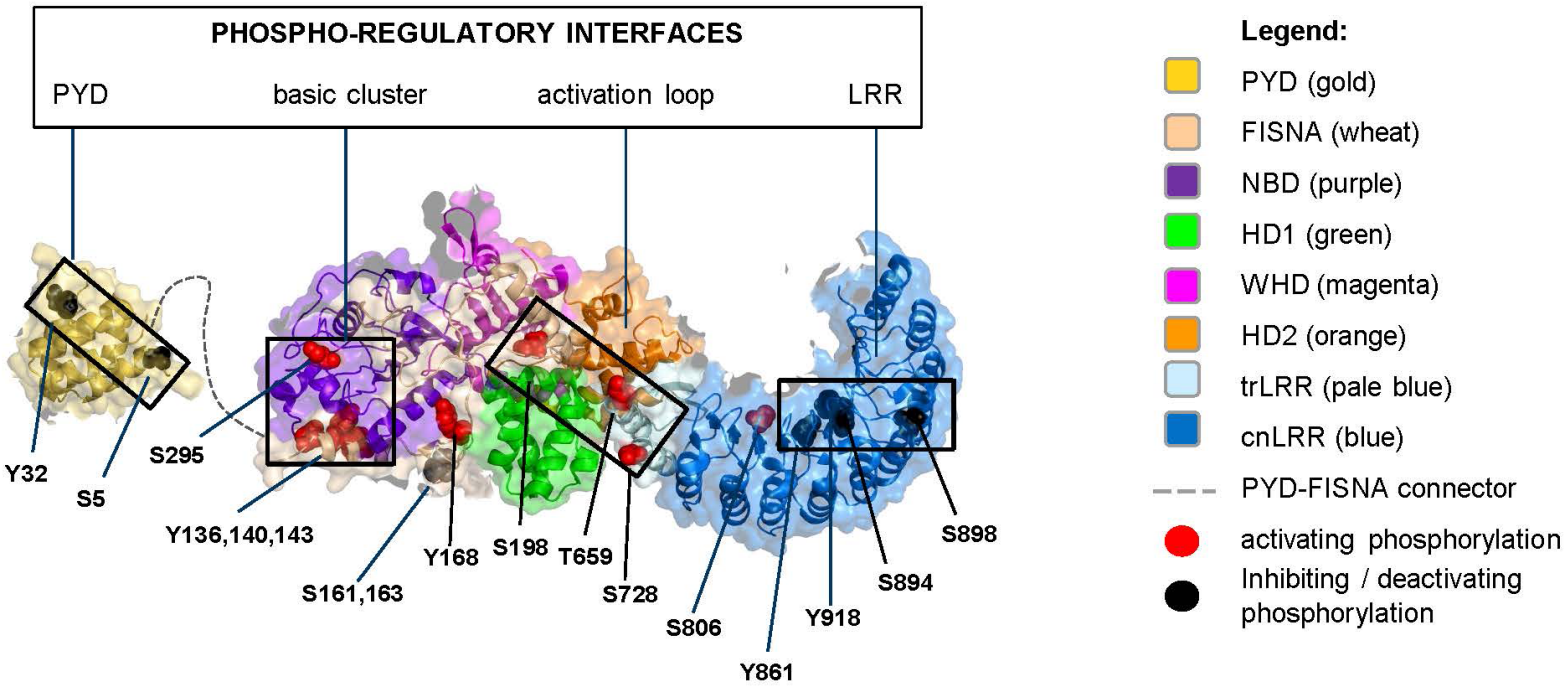
Monomeric NLRP3 (domains color boxed as in **Figure 2**) with annotated phosphorylation sites (color coded as in **Figure 2**).

Here, we will summarize the localization of the reported phosphorylation sites that can be retrieved from the available NLRP3 structures with the aim to evaluate their exposure and address possible evidence of critical interfaces during the regulation of the NLRP3 maturation process (**Figure 3**).

### 3.1 At a glance

**Figure 4:**
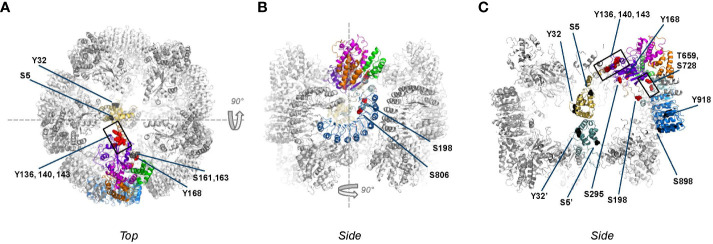
Phosphorylation sites mapped onto inactive, decameric NLRP3 (PDB:7pzc) in which one NLRP3 molecule is color-coded by domain (see **Figure 2** for code), with all other molecules of the complex shown in grey. Phospho-sites that are solvent accessible are shown by either red or black spheres. Please note that in this structure only two PYD domains are visible, one of which is shown in gold as part of the color-coded chain. **(A)** Top-view of the decamer, revealing the exposed helix containing Y136, Y140 and Y143 and the lack of interactions between adjacent NACHT domains; **(B)** side view obtained by 90 degrees rotation around the dashed line as shown in **(A)**; **(C)** slice-through of the side view after a 90 degrees rotation around the dashed line as shown in **(B)**. In this view, the two interacting PYDs are visible in the center of the decameric cage with the PYD of the opposite chain colored in teal for clarity, showing that the phospho-sites of the two PYDs are not interacting.

**Figure 5:**
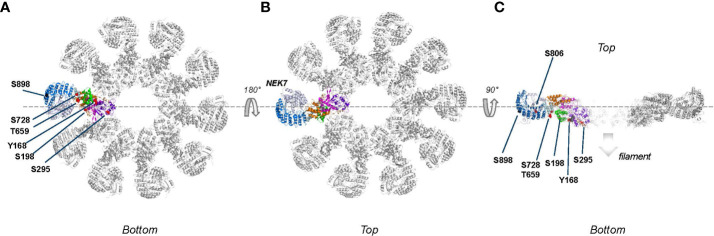
Phospho-sites mapped onto the active, fully assembled NLRP3 inflammasome (PDB:8ej4) that includes NEK7 (colored blue grey) bound to the LRR domain. Phospho-sites that are solvent accessible are shown by either red or black spheres. **(A)** ‘Bottom’ view, defined as the ASC fibers assembly side, from this side several phospho-sites are solvent accessible and therefore potentially accessible for modification; **(B)** Top view, obtained after 180 degrees rotation around the dashes lines as shown in **(A)**. Since NEK7 is masking the phosphosites on the concave side of the LRR domain, none of the described phospho-sites are clearly accessible from the top; **(C)** Slice through of view B after a 90 degrees rotation around the dashed line. The S806 site, which is not accessible when NEK7 is bound, is pointed with a dotted arrow.

All reported phosphorylation sites, with exception of those featured by the basic cluster, are within the buried interior of the inactive NLRP3 oligomer (**Figure 4**). Accessibility of the basic cluster for regulation by phosphorylation is consistent with the observation that, in resting conditions, about 50% of NLRP3 is reported to be tyrosine phosphorylated (52–54). In the active conformation, most phosphorylation sites appear to localize to the “bottom” face of the NLRP3 ring, from which the PYD/ASC filament grows, a notable exception being the S806 site, which is exposed on the upper face where NEK7 binds (**Figure 5**). These observations indicate the importance of modulating the reactivity of key functional interfaces of the NLRP3 protein, such as the PYD, the basic cluster, the activation loop, and the LRR.

### 3.2 The PYD interface *(S5, Y32)*

The structure of the PYD domain has a well-defined fold and has been visualized as part of the inactive decameric cage as well as the ASC-PYD filament. In the NLRP3 decamer, two PYDs were modeled inside of the cage, shielded from the outside (38); in the dodecameric cage residual density showed the presence of multiple PYDs yet these were not part of the final model (51). The S5 phosphorylation site is at the base of the first helix of the PYD (**Figures 3**, **4**; **Supplementary Figure 1**). S5 was proposed to reside at the PYD-PYD interface based on modeling studies derived from the ASC^PYD^ filament structure (68). The recent structures of PYD filaments (PDB: 7pzd and 8ert) show S5 to be located at the PYD-PYD interface formed by Helix A of one PYD and Helix B of the adjacent PYD. This interface seems to be stabilized by a salt bridge between the R7 and both E30 and D31, and phosphorylation of the nearby S5 would be spatially incompatible with the tight packing of PYDs in this filament (**Supplementary Figure 1**). This is in line with the studies showing that phosphorylation of S5 inhibits NLRP3 activation by blocking NLRP3 oligomerization as well as assembly with ASC (27). In the inactive decameric structure S5 is on the surface of each of the two resolved PYDs yet no interaction can be observed with neither PYD nor NACHT domains and we cannot conclude anything about the effect of S5 phosphorylation in the decamer structure (**Figure 4**). Of note, by preventing PYD-PYD interactions, phosphorylation of S5 likely increases the solubility of the PYD domain.

Another important phosphorylation site in the PYD is Y32, which resides at the tip of helix A2 (**Figures 3**, **4**). Like S5, the main impact of Y32 phosphorylation may be the charge effect that aids the solubility of monomeric NLRP3. In contrast to S5, in the inactive decamer (**Figure 4**), Y32 seems not involved in any PYD-PYD interaction and given its location in a surface exposed loop, there may be space for a phosphoryl group without affecting the packing inside the cage. Yet, as for S5, Y32 phosphorylation was reported to prevent ASC recruitment (32). This suggests differential impacts of these phospho-sites on the conformation of the PYDs.

How phosphorylation of S5 prevents the ubiquitylation of K496 (30) is not clear from the available structures. In the decameric and hexameric structures, S5 is buried inside the cage, whereas K496 is on the surface on the outside. Presumably, the NLRP3 cage interacts with lipids via the positively charged polybasic top face and in doing so it shields K496, thus protecting NLRP3 from TRIM31-mediated degradation. If phosphorylation of S5 were to have a direct stabilizing effect on NLRP3 by preventing K496 ubiquitylation, this potentially happens when NLRP3 is in its monomeric state.

Taken together, phosphorylation of the PYD domain (at S5 and/or Y32) on monomeric NLRP3 might add to its stability by introducing charge and potentially mediating PYD-NACHT interaction; phosphorylation of the PYD domain within the caged structure may or may not have an effect; phosphorylation of the PYD domain in the context of the fully assembled NLRP3 will interfere with the PYD-PYD interaction of the PYD-ASC filament and, by destabilizing the filament could potentially add to the disassembly of the inflammasome (51).

### 3.3 The basic cluster interface *(Y136, Y140, Y143, S161, S163, S295)*

The tyrosine phosphorylation sites Y136, Y140, and Y143 in the first helix of the FISNA subdomain are embedded in a basic cluster required for binding to membranes, which is exposed on the surface of the inactive NLRP3 assembly (**Figure 4**). The current hypothesis is that phosphorylation in this region may attenuate the capacity of NLRP3 to bind to negatively charged lipids on the membrane, leading to membrane dissociation of NLRP3 as a required step during the inflammasome activation process. In addition, this region was proposed to contribute to the PYD-PYD interaction between NLRP3 and ASC (35, 69). Upon phosphorylation, the basic cluster may be pushed away from the NACHT domain, thereby giving more flexibility to the connecting PYD domain. The effect of NLRP3 Y136 phosphorylation on activation is controversial. EphA2-mediated phosphorylation of Y136 (Y132 in mouse) is reportedly inhibitory whereas phosphorylation of the same residue by BTK (together with Y140 and Y143) is linked positively to NLRP3 activation. Phosphorylation at this site might confer distinct impacts on different paths leading to NLRP3 activation and/or at different stages of the NLRP3 activation process.

The potential phospho-sites S161 and S163, though never studied in detail, are noted by several studies (27, 32, 39, 40). These sites belong to a long loop of the FISNA domain, connecting the first helix of the domain, which contains the basic cluster, with the central beta sheet of the NBD subdomain. In the EM structure, these residues are resolved, while the region 152-160 is flexible. In the crystal structure, the entire region 152-164 is flexible and not resolved. S161 and S163 both form H-bonds with nearby residues (R237 and K166), thereby giving this flexible region local rigidity. In the decamer, these residues are solvent exposed. Their phosphorylation might affect local rigidity. Mutagenesis of these sites, however, did not underscore a critical impact (27, 39, 40).

Serine residue S295 lies in the center of the NBD domain, at the NACHT/NACHT interface. It is solvent exposed within the interior of decameric NLRP3 (**Figure 4**) and away from ADP yet could potentially stabilize the nucleotide pocket via phospho-S295-R252 interaction to affect ATP hydrolysis (**Supplementary Figure 2**). Phosphorylation of this site by PKA inhibits the assembly of the inflammasome complex, whereas phosphorylation by PKD at MAMs results in dissociation of NLRP3 from MAMs to allow for inflammasome assembly. Therefore, this site may need to be accessed at different stages of the NLRP3 licensing process. Importantly, helix 285-295 in which S295 is located faces the basic cluster helix. The structural impact of tyrosine phosphorylation at Y136/140/143 within this helix (see above) may facilitate accessibility to S295 for phosphorylation by PKD. In the active conformation S295 is not observed to be phosphorylated, but it is surface exposed and located at the interface with a neighboring molecule formed by the first helix of the FISNA. Since this helix contains positively charged residues K139 and K142, it is conceivable that phospho-Ser295 could interact with these residues to stabilize this ring interface. Whether phosphorylation by PKA of S295 on the active ring might induce disassembly of NLRP3/ASC complexes as suggested earlier remains unresolved (43).

### 3.4 The activation loop interface *(Y168, S198, T659, S728)*

Tyrosine 168 is visible in both the EM and Xray structures. Its sidechain faces the flexible 190-210 loop region (activation loop) (**Supplementary Figure 2**). Adjacent to Y168 is T169, which interacts with the nucleotide. Phosphorylation of Y168 may therefore regulate nucleotide binding (ATP/ADP) and/or ATP hydrolysis (35).

Serine 198 lies in the activation loop of NLRP3, upstream of the NBD domain. This loop region (180-198 in the EM decamer, 176-201 in the Xray) interacts with the LRR domain, which is hidden in the inner side of the NLRP3 barrel, further acting as a barrier to prevent premature activation. In fact, S198 is visible in the EM structure, very close to the first helix of the LRR domain (710–720) (**Figure 4**). Thus, phosphorylation of S198 might affect the interaction between the NBD and LRR domains and as a result, the relative orientation of these domains with respect to each other. It was found to be phosphorylated in the purified, baculovirus-expressed multimeric NLRP3 protein obtained by Hochheiser et al. (38): The peak showing enrichment in S198 phosphorylation coincided with a higher ATP hydrolysis rate, which suggested it is not in a fully blocked conformation despite its inability to act as a seed for inflammasome activation (38, 70). The latter finding further suggested that phosphorylation of S198 is not sufficient or that this site might require subsequent dephosphorylation to allow for further licensing. This is in line with the observation that priming was still required for activation of a S198D mutant (39). S198 may be positioned in a way similar to S533 in NLRC4 (71) and be able to interact with the LRR domain. Consistent with a key role of the activation loop, phosphorylation of T193, T195, and S201 was also reported beyond that of S198 (32, 38).

Threonine 659 belongs to the transition LRR, in the vicinity of an important binding interface with NEK7 (interface II) (72). Reconstitution studies with variants preventing phosphorylation at this site showed impaired interaction with NEK7 (40). Serine 728 also belongs to the transition LRR, downstream of the acidic loop. Phosphorylation of this site may support self-association of NLRP3 (50). This site is part of exon 5, which is known to be spliced out in a reported NLRP3 variant that lacks the capacity to bind NEK7 (73). Of note, T659 and S728 in the transition LRR are in the vicinity of S198 in the activation loop, supporting a key role of phosphorylation in this region to regulate the NACHT-LRR interface and impact on the NLRP3 cage dynamics (**Figure 4**).

### 3.5 The LRR concave (F2F) and convex (B2B) interfaces *(S806, Y861, S894/898, Y918)*

Serine 806 is close to positively charged residues from the adjacent LRR (*e.g.*, R1009). Therefore, its phosphorylation may stabilize the NLRP3 barrel and support TGN dispersion. It is located at the NEK7-binding interface on the concave surface of LRR, which explains why its dephosphorylation during NLRP3 activation is required to allow for NEK7 binding (49). Tyrosine 861 maps to the same area as S806 in the inactive NLRP3 cage structure and participates in LRR-LRR interactions. However, contrary to S806, its phosphorylation may target NLRP3 for sequestration into phagophores, the precursors to autophagosomes (54).

S894 and Y918 are also located on the concave site of LRR which constitutes the NEK7 binding site. Phosphorylation of Y918 down-regulates NLRP3, suggesting that post-translational modifications of residues in this region may directly interfere with NEK7 binding. This is consistent with the recent postulation that the role of NEK7 is to disrupt the NLRP3 cage to allow active disc formation (67, 74). In contrast, S898 is located on the convex site of the LRR domain and not in proximity of other domains or neighboring molecules, neither in the decameric cage nor in the active inflammasome. Hence the effect of its phosphorylation cannot be explained in structural terms (**Figure 4**).

Remarkably, all phosphorylation reactions reported at the LRR interfaces are inhibitory (phosphorylation at S806 inhibits NEK7 binding but is required during priming). The LRR domain has the propensity to self-oligomerize (resulting in cage formation) and to be involved in protein-protein interactions (*e.g.*, with NEK7). Therefore, akin to the PYD domain, preventing or limiting protein-protein associations may be key for tight control of inflammasome activation.

## 4 Playing the NLRP3 score: deciphering the sequence of phosphorylation events

**Figure 6:**
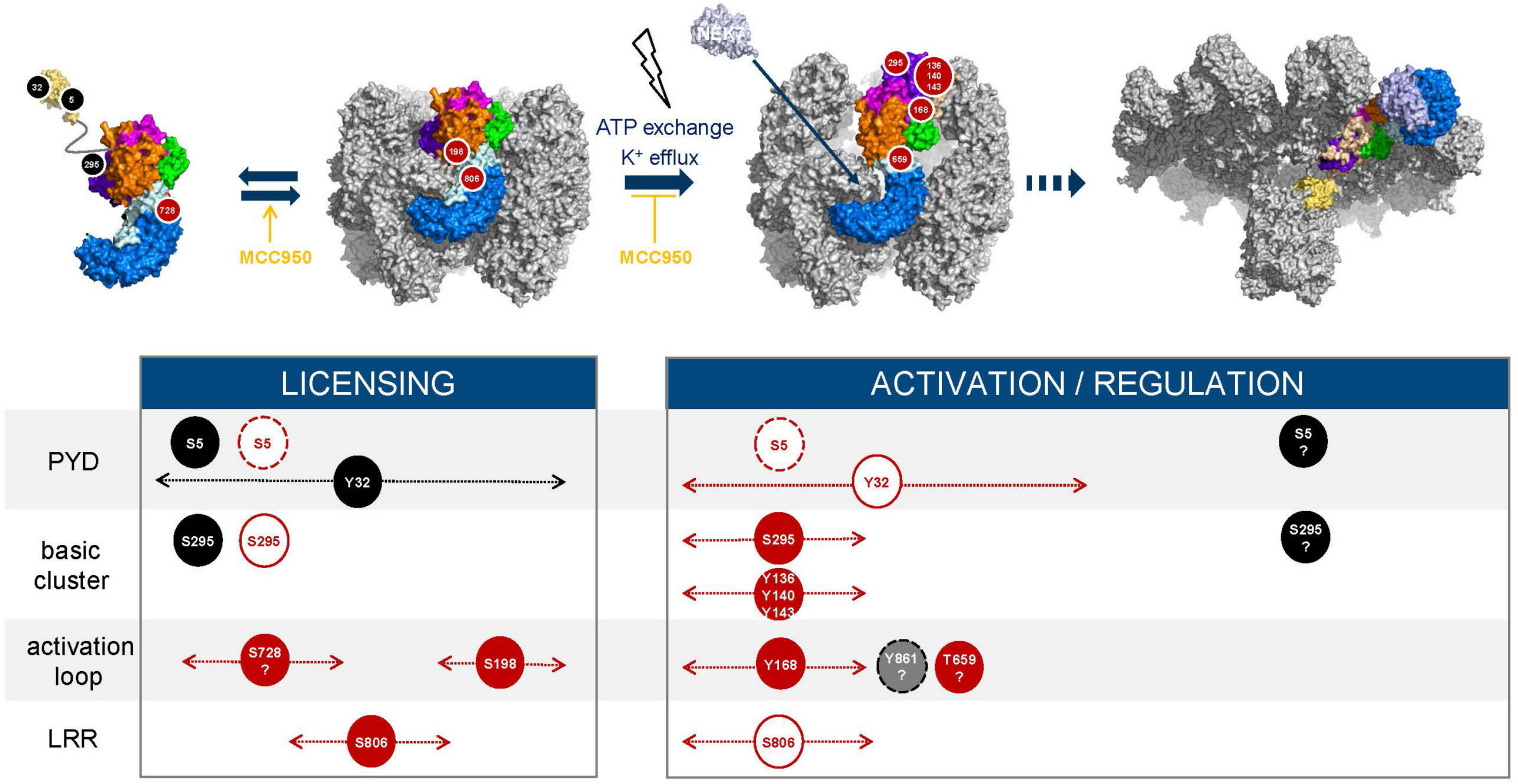
Phosphorylation timelines. Inhibitory (black) and activatory (red) events are displayed; phosphorylated residues (filled circles, black, red, grey) and dephosphorylated residues (white circles). The width with dotted lines and arrows represents the time frame during which an event may occur. Dashed circle lines indicate that the labelled event triggers degradation of NLRP3. Question marks refer to hypothetical events based on current reports and hypotheses. The NLRP3 inhibitor MCC950 freezes NLRP3 cages in their inactive, closed conformation (76–78) and increases or enhances the conversion of monomeric NLRP3 into membrane bound caged and inactive NLRP3 (74).

Since the pioneering findings by Fernandes-Alnemri et al. (75) showing that TLR signaling orchestrates an IRAK1-mediated, ROS-dependent post-translational program regulating NLRP3, several licensing events have been identified. Phosphorylation and dephosphorylation reactions are switch-like mechanisms to control NLRP3 and they involve several protein-protein interactions that need to be coordinated. In this section we have integrated the current knowledge to try and map out the sequence of phosphorylation and dephosphorylation events from the initial synthesis of NLRP3 until its assembly into an active inflammasome (**Figure 6**).

Phosphorylation by AKT at S5 likely represents an early regulatory step that controls both stability of NLRP3 monomers and their ability to assemble into cages at the membrane. Dephosphorylation by PP2A is required to allow for the inflammasome activation process and licenses NLRP3 for TRIM31-mediated degradation. Thus, phosphorylation of S5 may prevent early degradation of the newly synthesized NLRP3 monomer and may provide a brake restraining NLRP3 cage formation and assembly in the absence of an activation signal. Available data suggest that a balance between AKT and PP2A levels/activity may govern the amounts of caged NLRP3 available and that both enzymes are active/activated during priming, *e.g.*, following treatment with LPS. BTK may contribute to keeping NLRP3 activation in check by phosphorylating and thereby inhibiting PP2A which in turn limits dephosphorylation of S5. S5 is also surface exposed in the NLRP3 filament structure (67) and thus, could potentially be re-phosphorylated. Phosphorylation at a later stage of the activation process might serve the purpose of either preventing membrane binding after activation has occurred or limiting the size of the developing NLRP3 inflammasome filament to influence the extent of specking at the microtubule organizing center (MTOC).

Phosphorylation at Y32 is induced by LPS whereas dephosphorylation by PTEN, which is key for subsequent interaction with ASC, is triggered by nigericin (32). This mechanism may ensure that recruitment of ASC only comes at the right point in time or at the right location after activation has been triggered. At what stage phosphorylation occurs during licensing of NLRP3 before activation is unknown as well as whether phosphorylation at Y32 and that at S5 might, at least partially be fulfilling the same goal, *i.e*., could be partially redundant.

Serine 198 (and S201) in the FISNA activation loop is hidden in the inner side of the decamer, in close proximity to the outer helix α_L_, which follows the acidic loop in the LRR domain. This may represent another protection mechanism against premature activation. How the site becomes accessible for phosphorylation by JNK1 during the priming step remains to be elucidated; binding to membranes might be one parameter. Phosphorylation of S198 has been associated with high molecular weight NLRP3 complexes (larger than decamers) and ATPase activity (not detectable in decamers), which suggests a role of this phosphorylation in ATP exchange or in contributing to a change in the relative orientation of the NBD and LRR domains (38). Activation of NLRP3 by, *e.g*., nigericin triggers its deubiquitylation in a BRCC3-dependent manner (11, 13). Phosphorylation at S198 is required for interaction of NLRP3 with BRCC3 (39). Furthermore, NLRP3 needs to be dephosphorylated at S806 to interact with and be deubiquitinated by BRCC3, and interaction of NLRP3 with NEK7 is required for subsequent BRCC3 recruitment and deubiquitylation of NLRP3 (49). These observations are consistent with sequential phosphorylation of S806 and S198 during priming (**Figure 6**). Phosphorylation of S806 may push the trLRR towards opening, which then may require phosphorylation at S198 to adopt the fully open conformer. Activation would subsequently lead to dephosphorylation at S806. The conformational switch of the FISNA domain induced by potassium efflux (79) and the impact it produces on the NLRP3 barrel, in particular the nucleotide site (38) likely provides opportunities for further post-translational modifications, including perhaps the fate of the phosphate group at T198, which is still unresolved.

Phosphorylation at S806 occurs during priming as shown upon treatment with various TLR regimens (49). It has been suggested to stabilize the NLRP3 cage by interacting with R1009 on the adjacent LRR, which may facilitate membrane recruitment and support TGN dispersion (51). Preventing phosphorylation at this site abolished steady state phosphorylation at other serine residues in NLRP3, suggesting a key regulatory function (49). When exactly S806 needs to be dephosphorylated to allow for subsequent activation is not known.

Phosphorylation of several tyrosine residues, Y136, Y140, Y143 (BTK-mediated) upon activation is believed to reduce the affinity of NLRP3 for membranes due to charge. The three phosphosites likely affect the packing of helix ax (127–137) against the NBD which may provide the necessary flexibility to allow the PYD to exit from the decamer cavity during the transition from closed to semi-open decamer, a transition state that has not been observed as such but was suggested to happen when ATP transforms the NACHT domain of NLRP3 while still being in the decameric or dodecameric cage (51, 67). In addition, phosphorylation of Y168, which is also BTK-mediated, may impact nucleotide binding or the exchange process.

Phosphorylation of NLRP3 monomers at S295 by PKA may prevent cage formation and binding to membranes such as MAMs. Following dephosphorylation, re-phosphorylation of NLRP3 S295 by PKD has been proposed to result from approximation of PKD and oligomerized NLRP3 following stimulation-induced and DAG-mediated enrichment of PKD at the Trans-Golgi membrane network. Because S295 phosphorylation was reported to inhibit the ATPase activity of NLRP3 (43), it might also contribute to the lifespan of the active NLRP3 conformation (**Supplementary Figure 2**).

Further potential sites have been suggested but characterization remains too limited to understand when phosphorylation/dephosphorylation may occur and how it might fit in the sequence of events. Beside phosphorylation of S5 and S295, phosphorylation by TBK1 and IKKe downstream of TLR activation, at sites that remain to be identified might be another way to introduce a brake preventing premature activation of NLRP3 without a bona fide activation signal.

The reported interactions between domains in NLRP3 and their regulatory partners may also shed light on the sequence of the overall process. AKT was shown to bind constitutively to the LRR domain of NLRP3 via its kinase domain (80) and therefore may be poised for phosphorylation of S5, which as described above, is a key early post-translational event. Phosphorylation of S728 by MINK may also be an early event suggested to regulate the dimerization of NLRP3 and requiring interaction of MINK with the LRR domain of NLRP3 (50). BTK binds NLRP3 constitutively (35). It was also reported to bind to ASC (36). The determinants in NLRP3 are not known but BTK was able to phosphorylate a truncated construct lacking the PYD domain or the LRR domain, suggesting these two domains are not required for binding to BTK (35). Thus, BTK might directly bind to the NACHT domain, on a site that becomes accessible upon stimulation when the NLRP3 cage becomes less condensed. Similarly, PTEN may bind to NLRP3, via its phosphatase and C2 domains (32), upon exposure of the NACHT domain. PKD could phosphorylate S295 in NLRP3 lacking the LRR domain, suggesting this domain is not required for interaction with PKD (47). As above, PKD may access NLRP3 upon opening of the barrel. The PTPN22 phosphatase that dephosphorylates Y861 in the LRR domain needs ASC to interact with NLRP3 (52, 53), which indicates this dephosphorylation may take place at the assembly step, *i.e.*, late in the NLRP3 maturation process. The domains of interaction between NLRP3 and JNK1 and between NLRP3 and CK1α are yet undescribed (39, 49).

With the increasing understanding of potential subcellular locations of NLRP3, an emerging hypothesis is that NLRP3 may be present at different organelles, able to sense local triggers for activation (1). Consequently, there might be various interplays of kinases and phosphatases that regulate NLRP3 depending on its location. For instance, the mitochondria/ER/Golgi space has long been suggested to be an important compartment where NLRP3 accumulates and assembles upon activation (81). In particular, a role for microtubule was proposed to allow for a timely apposition of ASC on mitochondria to NLRP3 on the endoplasmic reticulum following activation (82). In an independent study, mitochondria were shown to act as a hub for assembly of the inflammasome complex. Upon priming with TLR ligands, NLRP3 and caspase-1 are recruited to cardiolipin on the outer surface of mitochondria in response to reactive oxygen species. During activation, ASC is recruited to mitochondrial NLRP3 in a calcium-dependent manner, resulting in inflammasome assembly (83). According to the model of Zhang et al. (47), following phosphorylation by PKD at the mitochondria-associated ER membranes (MAMs)- TGN interface, NLRP3 is released into the cytoplasm for inflammasome assembly. Recent work, using live cell multispectral time-lapse tracking acquisition study indicated that NLRP3 is essentially cytosolic under resting conditions (84). Upon stimulation with nigericin, NLRP3 starts oligomerizing and transiently associates with mitochondria before it aggregates progressively at the TGN. This work showed that these two organelles become interconnected after stimulation, which may provide a basis for transfer of NLRP3 from mitochondria to the TGN/TGN38+ vesicles. Because of the symmetry of NLRP3 inactive cages, each cage has two faces exposing the polybasic region and the question remains whether one cage can simultaneously interact with two membranes, possibly acting as bridging device between organelles. Mechanistically, stimuli that trigger the NLRP3 inflammasome induce GSK3β activation and binding to NLRP3, facilitating recruitment of NLRP3 to mitochondria and transition to the TGN. GSK3β also phosphorylates PI4k2A in the TGN to promote sustained NLRP3 oligomerization. These observations contrast with the data from the Zhang et al. study showing that NLRP3 does not physically relocate to the Golgi but instead benefit from the approximation of MAMs and Golgi during stimulation for being further processed towards full activation. Nevertheless, if NLRP3 is equipped with direct binding capability to PI4P, which is enriched in TGN38^+^ vesicles, via its polybasic region (85), it remains possible that it binds directly to the membrane of these vesicles. With the progress in understanding resolution at an organelle level (86, 87), the contrasting observations reported in these studies might reflect different measures of the same mechanism. In addition, there is increasing evidence that the TGN should be considered as an organelle independent of the Golgi (87). Going in this direction, very recent studies have revealed that NLRP3 inflammasome activators lead to accumulation of PI4P in the endosome resulting in impairment of endosome to TGN trafficking, which is necessary for endosomal recruitment of NLRP3 and subsequent activation (63, 64).

The cellular distribution of relevant kinases and phosphatases is another aspect that may provide information to better understand NLRP3 regulatory mechanisms. A kinase atlas covering 85% of the human kinome and annotated to 10 cellular compartments was recently described (88). Several kinases reported to regulate NLRP3 were included in this work and their localization score are summarized in **Table 1**. AKT1, 2, and 3 are similarly distributed between cytoplasm and plasma membrane. This is consistent with the understanding that AKT may phosphorylate S5 early on, when NLRP3 is still a monomer, controlling its degradation and regulating the pool that can assemble as a barrel at the membrane. This phosphorylation and its compartmentalization may also ensure that NLRP3 will not relocate to a subcellular organelle, *e.g.*, mitochondria, inappropriately (31). The localization of PRKACA and PRKACB [both PKA isoforms are reported to be involved in the regulation of NLRP3 (43)] indicates that PRKACB is fully cytosolic whereas PRKACA distributes between cytosol and Golgi. The cytosolic localization of PKA is consistent with its proposed role in the phosphorylation of S295 to gate the conversion of NLRP3 monomers into barrel, akin to the role of AKT at S5. The prominent localization of the PRKACA isoform to the Golgi compartment might suggest a role at a later stage of the NLRP3 maturation process, perhaps akin to PKD (see below). In this regard, it is notable that both PRKAs and A-kinase anchor proteins have been reported to harbor a Two-Phenylalanines-in-an Acidic-Tract (FFAT) motif, which is recognized by the VAP (VAMP-associated proteins) family of transmembrane proteins from the ER, involved in tethering other organelles (89, 90). The two isoforms of PKD, PRKD2 and PRKD3, reported to regulate NLRP3 (47, 48), share the unique feature among NLRP3 kinases, of vesicular localization, in addition to Golgi localization. This is consistent with a prominent role of PKD in the regulation of NLRP3 at the MAM/endosome/TGN space (47, 63). Remarkably, the two kinases CK1α (CSNK1A1) and JNK1 (MAPK8), which allow for barrel formation and/or may promote or assist barrel opening upon ATP exchange, are almost entirely cytosolic. These kinases may be poised for recruitment by membrane bound NLRP3 at various subcellular locations. The localization pattern of IKKe (IKBKE) is also worth mentioning. Uniquely, IKKe, which has been reported to regulate the ASC specking capacity of NLRP3 (28), scores high in the “aggresome” compartment. This suggests IKKe might take part into an aggresome-like mechanism for NLRP3, such as that recently described involving HDAC6 (91), which could target activated inflammasome for down-regulation at the MTOC. Consistent with an aggresome type of mechanism, phosphorylation of Y861 during NLRP3 activation was reported to allow for ASC-dependent recruitment into autophagosome by interaction with SQSTM1, targeting NLRP3 for degradation (54). Some of the aggregates localized to phagophores and some were recruited to lysosomes, indicative of degradation in autolysosomes. In addition, localization of NLRP3 to lysosome was recently shown to involve the Ragulator complex and HDAC6, and to be important for NLRP3 function (92).

**Table 1:** Subcellular localization of NLRP3-regulating protein kinases.

Kinase	Localization Score									
	N	C	CS	PM	MI	GL	ER	V	CT	AG
AKT1	1	4		5						
AKT2	1	4		5						
AKT3	1	4		5						
PRKACA		5				5				
PRKACB		10								
PRKACG		10								
PRKX	2	8								
PRKD1	1			7		2				
PRKD2		5		1		2		2		
PRKD3		3		2		3		2		
CSNK1A1	1	9								
CSNK1A1L	1	9								
MAPK8	2	7		1						
IKBKB	2	5		3						
IKBKE		6								4
NEK7	5	5								
MINK1		5		3				2		
EPHA2		1		1		3		5		
BTK	1	5		4						
LYN		1	1	8						
GSK3B		9		1						

C, Cytosol; N, nucleus; PM, plasma membrane; MI, mitochondrion; ER, endoplasmic reticulum; GL, Golgi apparatus; V, vesicle; CS, cytoskeleton; CT, centrosome; AG, aggresome. data and annotations as in (88).

Finally, there might be additional regulatory levels, such as allosteric kinase activation upon interaction with NLRP3, as recently suggested for BTK (35, 93). This could provide a mean to engage kinase activities at the relevant location selectively, for optimal control of inflammasome activation/deactivation.

## 5 Theme and variations in the NLRP3 score

As exemplified in this review, the NLRP3 phosphorylation score is complex. The identification of numerous sites, potentially regulated by several kinases and phosphatases, does not imply they must always be involved and that a unique sequence of events governs the NLRP3 maturation process. In fact, the theme on the NLRP3 score is likely amenable to variations.

It is remarkable that S198, one of the key phosphorylation sites identified in human NLRP3, is not conserved in species such as rat and pig. However, in both species, NLRP3 displays a nearby serine residue, which is conserved in human NLRP3 (S201) and was previously found to be phosphorylated (38). This site, as for S198, localizes to the “activation loop” of NLRP3 and therefore might provide an alternative phosphorylation option to impact on the conformation of the loop during NLRP3 activation. Other sites in the activation loop, such as T193, which is well conserved and was also suggested to be phosphorylated (32), might provide additional avenues for regulatory input in this critical space.

Several stress pathways can lead to NLRP3 inflammasome activation. A distinction must be made between extracellular agonists, such as nigericin and ATP, which act fast, and particulate agonists, such as MSU or silica, which have a slower onset. Some regulatory mechanisms have emerged that may apply differentially according to which type of agonist is used. For instance, nigericin-induced NLRP3 activation can be suppressed by PGE2, in a PKA-dependent manner, whereas PGE2-induced suppression of MSU and silica responses is not PKA mediated and thus, may not involve phosphorylation of NLRP3 at S295 (43). Similarly, dependence on BTK kinase activity was less pronounced when using nigericin to stimulate NLRP3, as compared to stimulation with LeuLeu-O-Me or with imiquimod (35). Consistent with the BTK observation, reovirus-induced NLRP3 activation in epithelial cells was inhibited by EphA2, whereas nigericin-induced activation was not (33). Another example of differential phosphorylation according to the nature of the stimulus is that of Y918, which is phosphorylated in response to soluble stimuli only (56). This is consistent with the reported plasma membrane localization of Lyn, the identified Y918 kinase (**Table 1**).

Differential phosphorylation might also be a way to drive the inflammasome activation process towards various outcomes. For instance, phosphorylation of T659 by Pak1 induced by the bacterial virulence factor CNF1 led to secretion of IL-1β independently of Gasdermin D, which might rely on an alternative secretion path using plasma membrane ruffles that are formed when the Rac GTPase is activated by CNF1 (40).

A given phosphorylation site might be phosphorylated by more than one kinase during the NLRP3 maturation process. For instance, independent studies have shown that both PKA and PKD can phosphorylate S295 in human NLRP3 (43–45, 47), possibly at a different step and in different cellular compartments. It is not known whether additional multiple phosphorylation cycles at sites beyond S295 are involved during the functional maturation of NLRP3. The case of Y136 is intriguing. EphA2 was reported to be phosphorylated by EphA2 and BTK, with different outcomes (33, 35). EphA2 is a receptor kinase selectively expressed in epithelial cells and is enriched at the vesicular compartment (**Table 1**) (88). Another site to continue watching is S5, which may remain accessible throughout the maturation process and could provide further means to regulate the functional spectrum of NLRP3 activation (**Figure 6**).

As mentioned earlier, phosphorylation and ubiquitylation mechanisms of NLRP3 regulation are interconnected. Ubiquitylation-driven down-regulation of NLRP3 by Parkin was described and may represent a specific pathway for regulation of NLRP3 in dopaminergic neurons (94). Whether this mechanism is gated by a phosphorylation reaction remains to be evidenced.

Finally, several splice variants of human NLRP3 have been identified, which have so far received little attention (73, 95, 96). A major form that lacks exon 5 was reported to be inactive (73). Other variants, lacking exon 4, exon 6, or exon 7 were also reported (73, 95) as well as several variants recently identified in human primary monocytes, some of which expressed at abundant levels (96). Consequently, because they may lack reported phosphorylation sites (*e.g.*, S728 lies in exon 5, S806 in exon 6, Y861 in exon 7), it can be expected that the maturation of NLRP3 variants will be differentially impacted.

**Figure 7:**
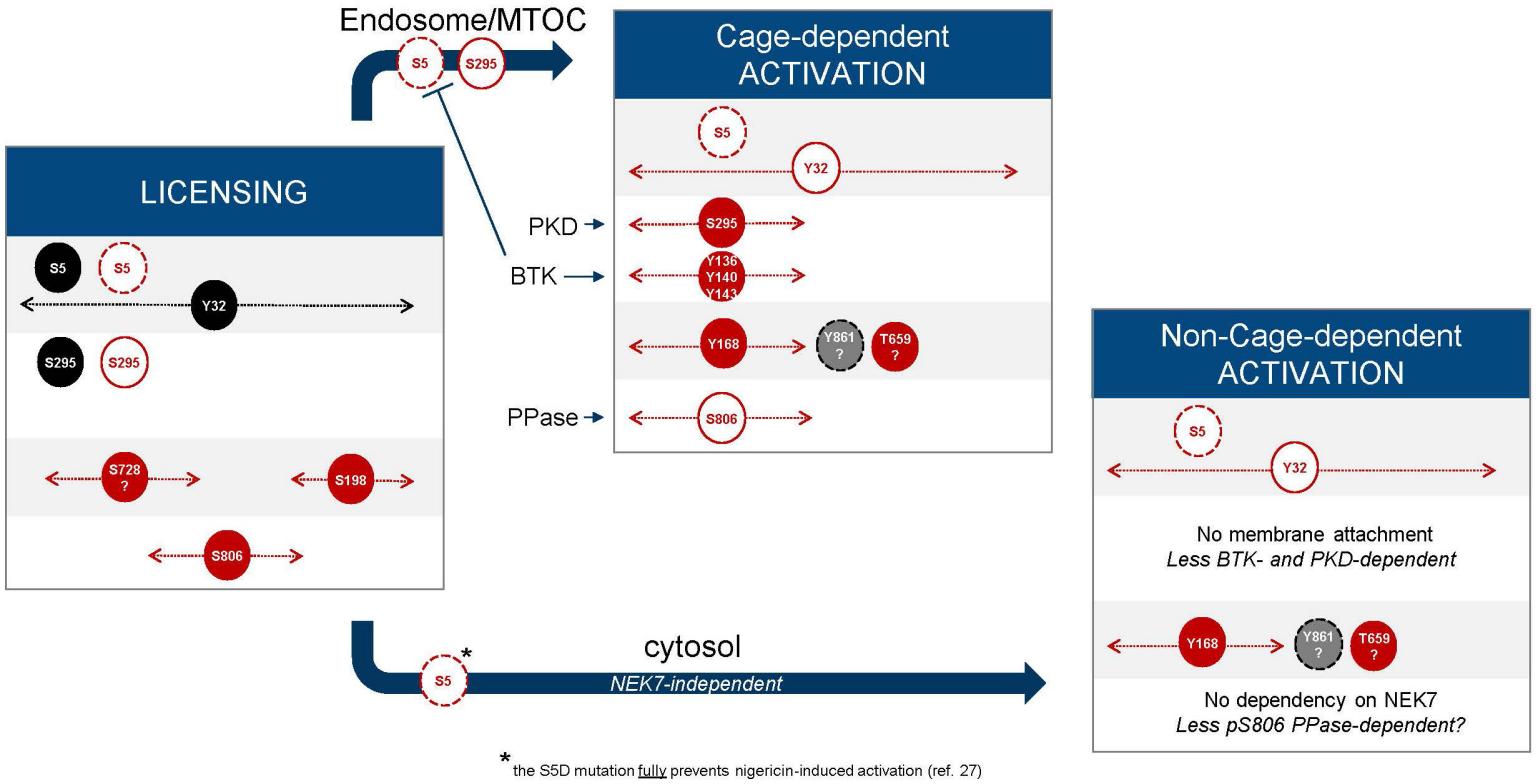
Cage-dependent and non-cage-dependent pathways. Extension from **Figure 6** with hypotheses, taking into account the recently described non-cage pathway, which might not rely on regulatory phosphorylation of the basic cluster based on available structural and biological evidence (74).

While the current knowledge of NLRP3 licensing is based on oligomeric cage formation, very recent data support the existence of an additional path, relying on cytosolic, non-cage forming NLRP3 species that do not traffic through the TGN and the MTOC (74). Remarkably, evidence was provided that the cage/TGN/MTOC dependent pathway is required for both K^+^-dependent (nigericin, MSU) and K^+^-independent (imiquimod) stimuli, whereas the non-caged pathway occurs only with the K^+^-dependent stimuli. Considering this, BTK, which is reportedly more relevant for NLRP3 activation by imiquimod compared to activation with nigericin (see above), might not be involved in the non-cage forming/monomeric NLRP3 pathway. Similarly, PKD impacts mostly the early phase of NLRP3 activation by nigericin (48) that is cage/TGN/MTOC dependent (74), and thus may be less critical for the non-cage activation pathway (**Figure 7**).

## 6 Perspectives

Different strategies have been used to obtain NLRP3 and identify putative phosphorylation sites. They may have led to stabilization of NLRP3/the inflammasome at various stages of the maturation pathway, resulting in the identification of several sites. One caveat of any approach is that the procedure could have artificially forced the system into a path that is not or is less physiologically relevant. Therefore, further validation work is needed before a robust roadmap of NLRP3 phosphorylation can be obtained and to confirm which kinases and phosphatases are involved.

For several sites, the bar in the score has been revealed but the orchestra players are incompletely known (**Figure 1**). For instance, the kinase section lead playing at Y32 or Y861 are not yet identified. For most sites the phosphatase section leads are also unclear. The bar containing S894 and S898, embedded in a phosphodegron motif also lacks both sections lead. Conversely, kinase section leads have been identified, such as IKKβ and TBK1/IKKϵ, but it remains unknown what part they may play (it might be in another score than NLRP3).

It is not well understood yet which phosphorylation sites/events are strictly necessary for controlling the complete NLRP3 maturation process ― NLRP3 protein stability, conformational changes, oligomerization, trafficking to and from membranal compartments, ATP binding and hydrolysis, inflammasome assembly ― and which ones might be less critical or amenable to compensatory mechanisms. To this aim, the further characterization of supramolecular organizing centers that may form and recruit kinases and phosphatases to coordinate responses during inflammasome activation is worth further work (97).

An important gap in the understanding remains whether post-translational mechanisms might influence the level of dependency on NEK7 of the NLRP3 maturation process, which seems to be variable between cells and species, and has remained elusive. As suggested already, there might be a degree of pleiotropy in the way NLRP3 can become functionally primed that requires further insights (65). Recent data suggesting an alternative, non-cage based, NLRP3 activation pathway, have proposed that NEK7 might only be involved in the cage-dependent activation process (74).

Several key questions remain to be answered. What is gating the capacity of NLRP3 to form a barrel at the membrane? What is the proportion of NLRP3 that may be present at organelles as sentinels in resting conditions and is phosphorylation playing a role in this? Upon binding to a membrane, is the cage becoming asymmetric? Could the opposite side of the barrel serve as an additional interface and engage in membrane binding? We speculate that such a scenario could assist membrane curving at the surface of an organelle and could contribute to approximation of two membranes/organelles, which might in turn allow for NLRP3 to traffic between organelles.

Further understanding of the organelle-specific interactome of NLRP3 including kinases and phosphatases should also clarify where the inflammasome can assemble to activate caspase-1. ASC specking activity is a widely used readout of inflammasome activation in cellular systems, which is believed to recapitulate inflammasome assembly and activation at the MOTC. However, recent work has shown that specks may also assemble independently of MTOC (74). Another study has suggested that the ragulator complex, assisted by HDAC6, can serve as a scaffold for lysosomal activation of NLRP3 whereas the HDAC6-mediated transport of NLRP3 to the MTOC may promote its targeting to autophagosomes for degradation (91, 92). Whether NLRP3 phosphorylation (*e.g.*, at Y861) and kinases (*e.g.*, IKKε) may differentially control these processes remains to be evaluated.

In conclusion, significant progress has been made to unravel the processes that contribute to NLRP3 functional maturation. Key studies have identified either NLRP3 residues that are post-translationally modified, quaternary structures that NLRP3 can adopt, or subcellular locations to which NLRP3 localizes during activation. The challenge ahead is to integrate these individual aspects of NLRP3 biology into a comprehensive model for stepwise NLRP3 licensing and activation. Structural information combined with subcellular information as well as interactome elucidation will undoubtedly help refine our understanding of the beautiful cellular machinery blossoming into an active inflammasome.

## Author contributions

FB: Conceptualization, Data curation, Methodology, Writing – original draft, Writing – review & editing. CD: Data curation, Software, Visualization, Writing – review & editing.
