# Supplementary material for: A phospho-harmonic orchestra plays the NLRP3 score
Source: Front Immunol. 2023 Nov 3;14:1281607. doi: 10.3389/fimmu.2023.1281607 (PMC10654991; doi:10.3389/fimmu.2023.1281607)
Supplement: Supplementary file 1 [file Presentation_1.pptx]

## Slide 1
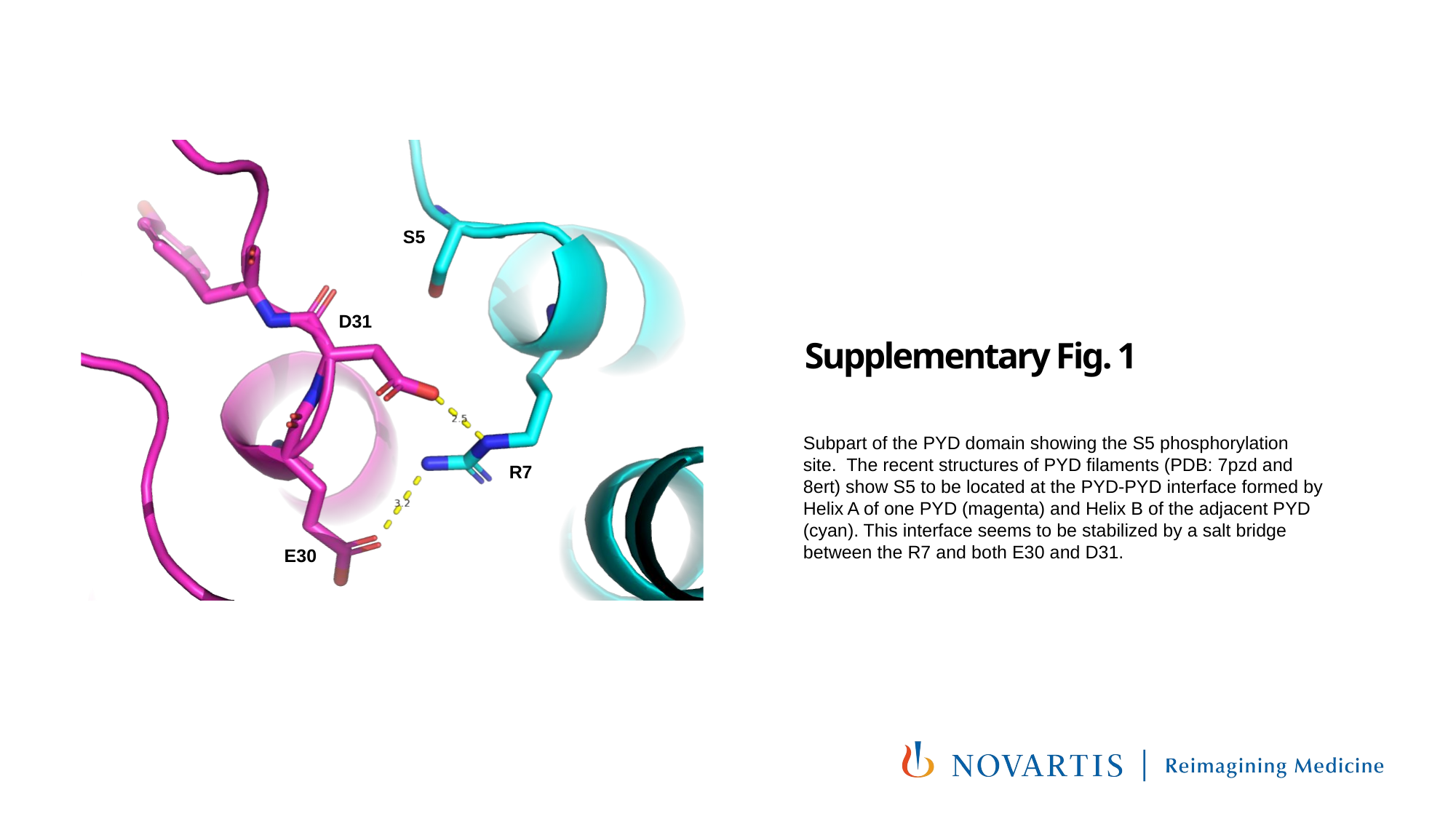

S5
D31
# Supplementary Fig. 1
Subpart of the PYD domain showing the S5 phosphorylation site. The recent structures of PYD filaments (PDB: 7pzd and 8ert) show S5 to be located at the PYD-PYD interface formed by Helix A of one PYD (magenta) and Helix B of the adjacent PYD (cyan). This interface seems to be stabilized by a salt bridge between the R7 and both E30 and D31.
R7
E30
