# Supplementary material for: A phospho-harmonic orchestra plays the NLRP3 score
Source: Front Immunol. 2023 Nov 3;14:1281607. doi: 10.3389/fimmu.2023.1281607 (PMC10654991; doi:10.3389/fimmu.2023.1281607)
Supplement: Supplementary file 2 [file Presentation_2.pptx]

## Slide 1
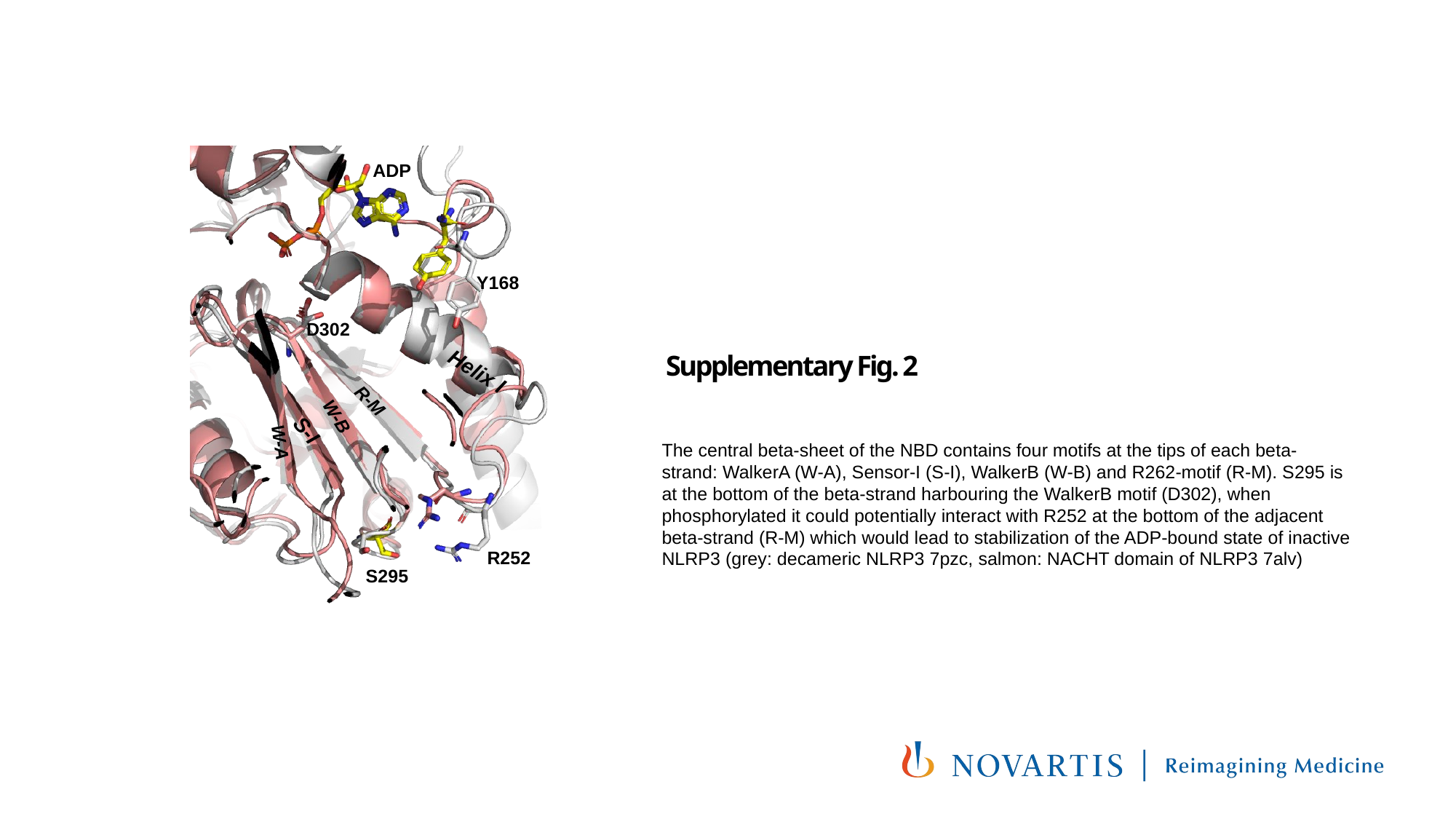

ADP
Y168
D302
Helix I
W-B
S-I
R252
S295
R-M
W-A
# Supplementary Fig. 2
The central beta-sheet of the NBD contains four motifs at the tips of each beta-strand: WalkerA (W-A), Sensor-I (S-I), WalkerB (W-B) and R262-motif (R-M). S295 is at the bottom of the beta-strand harbouring the WalkerB motif (D302), when phosphorylated it could potentially interact with R252 at the bottom of the adjacent beta-strand (R-M) which would lead to stabilization of the ADP-bound state of inactive NLRP3 (grey: decameric NLRP3 7pzc, salmon: NACHT domain of NLRP3 7alv)
